# Clubfoot Deformity Treatment with Ilizarov Apparatus in the Paediatric Population without Corrective Osteotomies and Soft Tissue Release: A Cross-Sectional Study

**DOI:** 10.5704/MOJ.2311.007

**Published:** 2023-11

**Authors:** HS Aslani, MB Athari, R Tavakoli-Darestani, A Pourmojarab, M Baroutkoub, M Zamani

**Affiliations:** 1 Department of Orthopedic Surgery, Tabriz University of Medical Sciences, Tabriz, Iran; 2 Department of Orthopedic surgery, Shahid Beheshti University of Medical Sciences School of Medicine, Tehran, Iran

**Keywords:** neglected clubfoot deformity, ilizarov apparatus, paediatric orthopaedics

## Abstract

**Introduction:**

Neglected Club Foot deformity is not an uncommon limb anomaly encountered by orthopaedic surgeons. Many treatment methods have been proposed. Ilizarov apparatus is one of the techniques used to correct this deformity.

**Materials and methods:**

In this cross-sectional study 47 patients (56 feet) between the ages of 5 and 10 years with clubfoot deformity were treated using the Ilizarov external fixator. Age, sex, type of deformity, and radiographic parameters were measured on foot radiographs. Also, the American Orthopaedic Foot and Ankle Society (AOFAS) score and the Dimeglio classification were recorded for each patient before and after treatment.

**Results:**

The treatment was unilateral in 38 patients and bilateral in 9 patients. 39 patients (69.6%) were male, and 17 patients (30.4%) were female with a mean age of 7.86 ± 1.4 years. Plantar angles of ankle flexion and ankle flexion curve increased from 20.12±6.52 and -16.51±8.36 to 25.89±6.44 and 6.19±6.42, respectively. There was also an improvement in the talocalcaneal and tibiocalcaneal angles. Also, the angle between the first metatarsus and the talus in the front and side views improved (P<0.00). Additionally, the mean AOFAS score and Dimeglio classification significantly improved. Three cases were complicated with distal tibial physeal separation that were treated with additional open surgeries.

**Conclusion:**

Ilizarov technique without osteotomies and soft tissue release could be considered a less invasive and successful method of treatment for neglected clubfoot deformity in patient five to ten years old that are not good candidate for Ponseti method.

## Introduction

First described by Hippocrates around 500 BC, clubfoot or congenital talipes equinovarus is one of the most common orthopaedic anomalies, with an incidence of one to two per 1000 live births^1^. This condition could occur isolated or in association with other serious congenital problems, particularly in severe and bilateral forms. The ideal therapeutic goal in these patients is to achieve a painless usable limb with a normal function for long-term walking movements^2^.

This anomaly will become a challenge for paediatric orthopaedic surgeons, considering the high recurrence rate, regardless of the surgical or non-surgical treatment strategy. One of the aetiologies of such a high recurrence rate is the surgeon's inability to identify the underlying pathoanatomy of the clubfoot. Other causes include muscle imbalance, soft tissue shrinkage, post-operative infections, and inadequate follow-up^3^. Clubfoot is usually considered an equinovarus deformity, but it should be noted that there are other hybrid conditions such as calcaneovagus, equinovalgus, and calcaneovarus. True clubfoot is identified by four characteristics: equinus, varus, adductus, and cavus^3,4^. The Ponseti method of casting is the gold standard for treatment of clubfoot deformity, and it is best started at the first week of life^5^. The neglected clubfoot is when patients don’t receive appropriate treatment until they start to walk^6^.

Treatment options of neglected clubfoot include both surgical and non-surgical techniques. Non-surgical techniques include Modified Ponseti technique for neglected clubfoot^7,8^. Ilizarov technique, extended release, triple arthrodesis, talectomy are surgical techniques available for neglected clubfoot deformity. Ilizarov technique with or without soft tissue release or osteotomy is a suitable method for the management and treatment of neglected and relapsed clubfoot deformity^9-11^. This method is less invasive compared to open surgical procedures and allows all deformity components to be corrected to some extent^12,13^. This technique has been modified and optimised by many authors over time. The less need for soft tissue release and osteotomy in the Ilizarov method gives it the clear superiority of maintaining the range of motion and leg length. Moreover, in the paediatric age range that most club foot cases are diagnosed, the foot still has the ability to remodel, and it is expected that in the long run, the function of the limb might be better than classical treatments^1^. Considering the gap in the evaluation of this treatment method and also the significant prevalence of club foot deformity, we decided to evaluate the results of using the Ilizarov technique without soft tissue release in the paediatric population.

## Materials and Methods

This study is a prospective multicentre cross-sectional survey conducted between March 2016 and April 2018 in Shohada university hospital in Tabriz, Iran, and Imam Hossein university hospital, Tehran, Iran. The study population was 47 patients (56 feet) between the ages of 5 and 10 years with clubfoot deformity. Inclusion criteria were: (1) confirmation of clubfoot using diagnostic criteria by two orthopaedic surgeons, (2) age between five to ten years, (3) no previous surgical or non-surgical treatment to improve clubfoot, and (4) patient’s and his/her caregiver's informed consent to use Ilizarov apparatus. Exclusion criteria were: (1) the presence of another major bony deformity in the foot other than clubfoot, (2) obvious muscle atrophy of the lower extremities, (3) systemic diseases involving bone metabolism, and (4) children with diagnosed genetic syndromes. We chose children between five and ten years old because the foot has time to grow and triple arthrodesis may not be the best option in this range and children younger than this age have more foot flexibility and could be managed through Ponseti casting method.

This study was designed according to the Declaration of Helsinki and approved by the Ethics Committee of Shahid Beheshti University of Medical Sciences and Tabriz University of Medical Sciences. Due to the young age of patients, while explaining the measures, expectations, and goals of treatment in simple language, written consent has been obtained from all caregivers of patients before starting treatment. During the consent process, the risks and possible side effects of treatment were explained to the participants. They were assured that no additional intervention would be performed and that all procedures performed were necessary. Participants were told that at any stage of the follow-up they would be able to withdraw from the study and that patient information was confidential and would not be disclosed at any stage of the investigation.

Age, sex, type of deformity, and angles of varus, equinus, adduction, and supination were examined pre-operatively by a trained orthopaedic surgeon. Talocalcaneal angle (on anteroposterior and lateral radiographs), Tibiocalcaneal (on lateral radiographs) and Talus-First Metatarsal angle were measured on foot radiographs^14^. The plantar flexion (Equinus) and dorsiflexion angle were measured by examination. The duration of the use of the Ilizarov apparatus and the follow-up of patients were also recorded for each patient. All patients were followed for a minimum of two years. The American Orthopaedic Foot and Ankle Society (AOFAS) score and the Dimeglio classification were also recorded for each patient before and after treatment. The Dimeglio classification utilises a 20-point scoring system by evaluating the residual deformity after applying gentle corrective manoeuvres. The severity of the deformity is then graded I-IV based on this scoring, a score of 1 to 5 for grade I, a score of 5 to 10 for grade II, a score of 10 to 15 for grade III, and a score of 15 to 20 for grade IV (Table I). The AOFAS score is also calculated based on a 25-item questionnaire. Variables such as pain, activity limitations, support requirement, walking surfaces, and foot motion are included in this questionnaire.

**Table I:** Patients clinical and radiological examination.

	Plantar Flexion	Dorsiflexion	Talocalcaneal (AP)	Talocalcaneal (LAT)	Tibiocalcaneal (LAT)	Talus First Metatarsal Angle (AP)	Talus First Metatarsal Angle (LT)
Pre-operative	20.12±6.52	-16.51±8.36	-19.91±6.06	-23.87±6.75	102.85±8.83	-19.21±5.50	-22.65±6.66
Post-operative	25.89±6.44	6.19±6.42	-1.35±7.98	-5.6±5.35	82.10±6.98	-1.42±7.61	-5.01±5.34
p-value	<0.05	<0.05	<0.05	<0.05	<0.05	<0.05	<0.05

Abbreviations - PF: ankle plantar flexion, DF: ankle dorsiflexion, Pr: preoperative, Po: postoperative, TC: talocalcaneal angle, TiC: tibiocalcaneal angle, TM: talus 1st metatarsal angle, f: female, M: male AP: anteroposterior, Lat: lateral, L: left, R: right, º: degree, AP: anteroposterior, LAT: lateral

**Fig 1:**
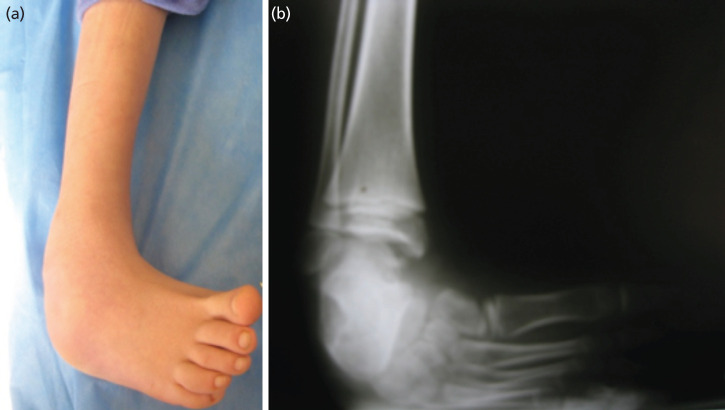
(a, b) Pre-operative radiograph and photograph of a patient with clubfoot.

**Fig 2:**
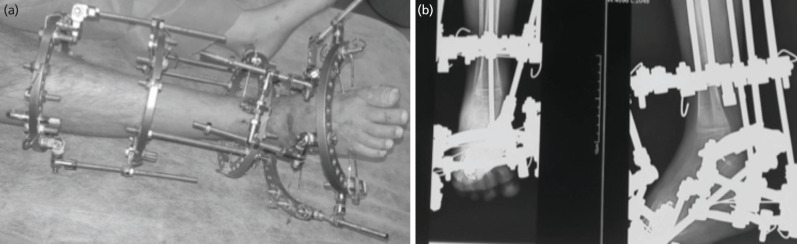
(a, b) Ilizarov Apparatus and post-operative radiographs.

All surgeries were performed by two surgeons (two of the authors) under general anaesthesia in the supine position. One to three full rings were mounted on the distal tibia using two tensioned wires for each ring. Fixation of the calcaneus was achieved using two olive wires (OW) in opposite directions. An OW was passed through the neck of the first and fifth metatarsal bones, and another proximal wire was added to the metatarsal wires. The calcaneus half-ring was connected to the tibial ring using three rods (one posteriorly and the other two on both sides). The half-hoops of the forefoot and calcaneus were connected using lateral and medial rods, and a plate hinge was placed between all the rods. Distraction was started at the second day after surgery. The patients were educated to distract the apparatus correctly in Hospital stay. For the hindfoot the medial rod connecting tibia and calcaneus was distracted at the rate of one millimetre per day and lateral rod at the rate of half a millimetre per day. For forefoot correction was achieved by distracting lateral side by one millimetre per day. The distraction was continued until the correction was achieved (the distraction method was taught to patients and supervised weekly by surgeons). After correction the Ilizarov apparatus was removed, and patient was put in a short leg walking cast for month. Fig. 1 and 2 demonstrate pre-operative and post-operative of a patient undergone treatment.

Kolmogorov-Smirnov test was used to evaluate normality. If its result indicated abnormal distribution, appropriate non-parametric tests were used. To modify confounding variables general linear models were utilised and to describe descriptive findings, frequency, percentage, mean, and standard deviation were used. We used paired t-test to compare the quantitative findings before and after treatment and the Wilcoxon Signed Ranks Test to analyse qualitative data before and after treatment. All statistical tests were performed using SPSS software version 19 [IBM Corp. Released 2010. IBM SPSS Statistics for Windows, Version 19.0. Armonk, NY]. The P-value of less than 0.05 was considered significant.

## Results

In this study, 47 patients (56 feet) were evaluated. The treatment was unilateral in 38 patients and bilateral in 9 patients. A total of 39 patients (69.6%) were male, and 17 patients (30.4%) were female, and the mean age of patients was 7.86±1.4 with the minimum and maximum age of 5 and 10 years. The median age of patients was 8 years.

Changes in the measured angles before and after treatment, all indicated an improvement in the patient's condition. Plantarflexion angles of ankle dorsiflexion angle increased from 20.12±6.52 and -16.51±8.36 to 25.89±6.44 and 6.19±6.42, respectively. There was also an improvement in the talocalcaneal and tibiocalcaneal angles. Also, the angle between the first metatarsus and the talus in the front and side views improved. All recorded changes were statistically significant (P<0.05). Additionally, the mean talocalcaneal angle (Anteroposterior radiographs) increased from - 19.91±6.06 to -1.35±7.98° and the mean talocalcaneal angle (Lateral radiographs) increased from -23.87±6.75 to - 5.6±5.35°.

In our study, the mean AOFAS score significantly increased from 52.46±9.63 pre-operatively to 74.87±7.1 post-operatively (p<0.001). In the Dimeglio scoring system, before the interventions, the majority of patients were in class III (32.1%) and class IV (62.5%). After the treatment with the Ilizarov apparatus, 69.6% of patients were in class I and 30.4% in class II, and none were categorised as classes three and four.

**Fig 3:**
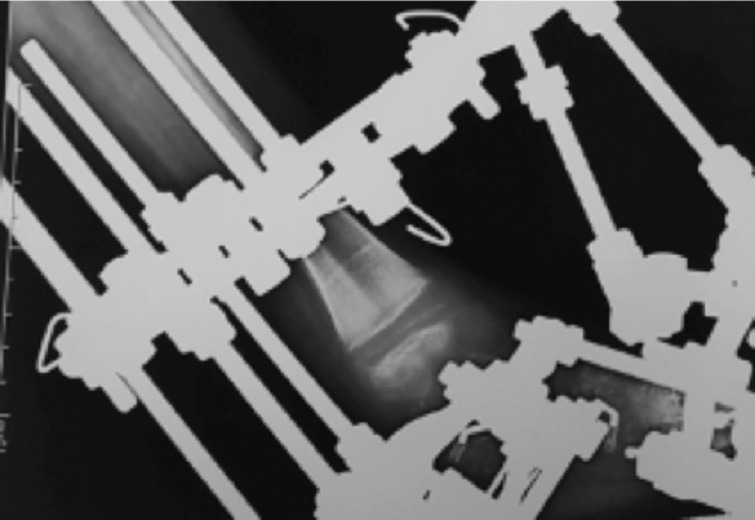
Physeal separation during treatment.

Of the four main characteristics of clubfoot deformity (equinus, varus, adductus, and cavus) equinus was the most resistant to treat using the Ilizarov technique in our cases. In three patients, this led to the physeal separation of the distal tibia (probably due to patient incompliance) in the follow-up, which required additional surgical interventions to treat. After diagnosing the physeal separation the distraction was stopped, and compression was started until the physis appeared normal on radiographs. After two weeks of rest the distraction was restarted. The patients with physeal separation had no physeal arrest at the end of the treatment. Other minor complication includes pin tract infection which treated by antibiotics and pin care and extraction of pins was not required (Fig. 3). The results are summarised in Table I.

## Discussion

In this study, we evaluated the results of using the Ilizarov apparatus without soft tissue release in paediatric patients aged 5-10 years with neglected clubfoot deformity. The therapeutic objectives in this study are, as previously stated, to get a pain-free functioning foot and to regain the capacity to walk easily in everyday shoes. Open surgical interventions are associated with unresolved deformity in 30% of cases and may lead to the recurrence of the disease^1^. Although the classic treatment for clubfoot at this age group is soft tissue release and osteotomy, repeated surgeries may end in a small, painful, and dry foot, and in cases that undergo bony interventions, shortening of the limb may occur. Other possible complications include restriction of foot movements and impairment of blood circulation^1,2^.

In the present study, out of the 47 patients (56 feet), 39 patients were male. Given the epidemiology of the disease and the 3 to 1 ratio of males to females, such a sex distribution is reasonable. In the study of Khanfour *et al*^15^, the mean age of patients at the time of surgery was 10.9 years. In our study, all the patients are less than 10 years old. We believe age plays an important role in determining the response to Ilizarov treatment, so this issue helps to increase the comprehensiveness of the results. All the measured changes in angles before and after treatment indicated an improvement in the patient's condition. Ankle flexion curves and the talocalcaneal and tibiocalcaneal angles, and the angle between the first metatarsus and talus in the front and side views, all recorded statistically significant changes.

In a study conducted by Hosny *et al*^10^, which is very similar to our research in terms of treatment, the procedure outcomes were reported qualitatively, with a good result in 20 and an acceptable result in 3 out of the 23 patients. In the study of Khanfour *et al*^15^ out of the 25 evaluated cases, 21 cases had good, and 4 cases had acceptable treatment results. Ferreira *et al*^16^ concluded that after 18 months follow-up period (12 to 107 months) 78.9% of their cases had acceptable results. Kocaoglu *et al*^17^ showed that at the end of two years of follow-up, out of a total of 23 patients with clubfoot, 21 cases had a plantigrade foot.

Concerning the quantitative outcomes of therapeutic options in the previous studies, Refai *et al*^18^ reported a significant improvement after treatment. In the study by Refai *et al*^18^, the mean AOFAS score was significantly increased compared to the amount measured at the initiation of the treatment (11 57 57 vs 18 81 81). In our study, these changes before and after treatment were 52.46±9.63, 52.46 and 74.87±7.1, respectively (p<0.001).

The Dimeglio classification was evaluated as an appropriate and acceptable criterion for determining clubfoot severity. Prior to treatment interventions, a large proportion of our patients were in class III (32.1%) and class IV (62.5%). After treatment, 69.6% of patients were in class I and 30.4% in class II, which is another evidence of the effectiveness of the treatment used in this study.

Complications-wise, out of the 56 feet we had 3 cases of distal tibial physeal separation during the course of the treatment. It is to be noted that all the mentioned three cases failed to complete the scheduled timely follow-up visits and had less-than-expected compliance. In the Ilizarov technique, the hindfoot equinus and varus deformity are corrected by applying gradual distraction from the distal tibial ring to the calcaneal ring. Frequent radiographic examination is essential to early diagnosis of any signs of physeal separation. All the three mentioned cases were transferred to the operating room and the distraction rods were removed and reversed. Additional open surgery for posterior talotibial joint capsular release and Achilles tendon lengthening (ATL) was required to treat the complication in these three cases with acceptable results.

One obvious limitation in our study is the relatively small sample size that could potentially render biased results, especially about complication rates. It is not obvious whether the three physeal separations encountered during our study could be prevented by closer follow-up visits or is an inevitable complication of this method of treatment that might be clarified with further larger studies. Other limitation in our study is Dimeglio system has been developed primarily for clubfeet in infant and new scoring system has been developed after we started our research^19^.

## Conclusion

Due to the complexities that exist in the nature of clubfoot deformity, the preferred choice for treatment depends on the condition of each patient and the opinion and ability of the surgeon. The use of the Ilizarov external fixator technique without the release of soft tissue or corrective osteotomies, as a less invasive method of treatment, has been associated with excellent therapeutic results, and the result of the current study once again reminds the importance of this treatment in the management of clubfoot patients. We believe that this method might also be successful in the treatment of patients with prior failed surgeries or recurrent clubfoot which seems to be a potential field for further studies. For the highlights, this article provides the results of the treatment of clubfoot deformity in 47 patients. The findings could be summarised and highlighted as follows: (a) Ilizarov external fixator is a minimally invasive method for the treatment of clubfoot deformity, (b) soft tissue release and osteotomies are not necessary for the correction of the deformity, (c) distal tibial physeal separation could occur as a known complication of this technique.
